# Cost effectiveness of option B plus for prevention of mother-to-child transmission of HIV in resource-limited countries: evidence from Kumasi, Ghana

**DOI:** 10.1186/s12879-015-0859-2

**Published:** 2015-03-18

**Authors:** Adam VanDeusen, Elijah Paintsil, Thomas Agyarko-Poku, Elisa F Long

**Affiliations:** Department of Chronic Disease Epidemiology, Yale School of Public Health, New Haven, CT USA; Departments of Pediatrics & Pharmacology, Yale School of Medicine, Yale Child Health Research Center, 464 Congress Ave, New Haven, CT USA; Department of Pharmacy, Kwame Nkrumah University of Science and Technology, Kumasi, Ghana; University of California Los Angeles, Anderson School of Management, Los Angeles, CA USA

**Keywords:** HIV prevention, Mother-to-child HIV transmission, Cost-effectiveness analysis, Mathematical model

## Abstract

**Background:**

Achieving the goal of eliminating mother-to-child HIV transmission (MTCT) necessitates increased access to antiretroviral therapy (ART) for HIV-infected pregnant women. Option B provides ART through pregnancy and breastfeeding, whereas Option B+ recommends continuous ART regardless of CD4 count, thus potentially reducing MTCT during future pregnancies. Our objective was to compare maternal and pediatric health outcomes and cost-effectiveness of Option B+ versus Option B in Ghana.

**Methods:**

A decision-analytic model was developed to simulate HIV progression in mothers and transmission (in utero, during birth, or through breastfeeding) to current and all future children. Clinical parameters, including antenatal care access and fertility rates, were estimated from a retrospective review of 817 medical records at two hospitals in Ghana. Additional parameters were obtained from published literature. Modeled outcomes include HIV infections averted among newborn children, quality-adjusted life-years (QALYs), and cost-effectiveness ratios.

**Results:**

HIV-infected women in Ghana have a lifetime average of 2.3 children (SD 1.3). Projected maternal life expectancy under Option B+ is 16.1 years, versus 16.0 years with Option B, yielding a gain of 0.1 maternal QALYs and 3.2 additional QALYs per child. Despite higher initial ART costs, Option B+ costs $785/QALY gained, a value considered very cost-effective by World Health Organization benchmarks. Widespread implementation of Option B+ in Ghana could theoretically prevent up to 668 HIV infections among children annually. Cost-effectiveness estimates remained favorable over robust sensitivity analyses.

**Conclusions:**

Although more expensive than Option B, Option B+ substantially reduces MTCT in future pregnancies, increases both maternal and pediatric QALYs, and is a cost-effective use of limited resources in Ghana.

**Electronic supplementary material:**

The online version of this article (doi:10.1186/s12879-015-0859-2) contains supplementary material, which is available to authorized users.

## Background

Global efforts to reduce mother-to-child transmission (MTCT) of HIV have made substantial progress with a 52% reduction in new infections occurring in children between 2001 and 2012, in large part due to improved access to antiretroviral therapy (ART) among pregnant women [1]. Despite this considerable progress, only 67% of pregnant women living with HIV in low- and middle-income countries received ART in 2013 (http://www.who.int/mediacentre/factsheets/fs360/en). The prevention of MTCT (PMTCT) of HIV in resource-limited countries is hindered by factors such as breastfeeding practices, inadequate healthcare infrastructure, potential stigma associated with not breastfeeding, and competing public health priorities in the face of limited healthcare resources. In June 2011, the United Nations General Assembly High Level Meeting on AIDS affirmed the Global Plan towards the elimination of new HIV infections among children by 2015 and keeping their mothers alive [2].

The World Health Organization (WHO) currently recommends two strategies to eliminate MTCT: Option B and Option B+. Option B, the current recommendation in Ghana, consists of antiretroviral prophylaxis that begins early in gestation and continues through breastfeeding for women with a CD4 count above 350 cells/mm^3^, and lifetime ART for women with a CD4 count below 350 cells/mm^3^ [3]. Option B guidelines have the potential to reduce rates of MTCT to as low as 1%, assuming high access to antenatal services [4]. Additionally, Option B can improve maternal health and is preferred to the previously recommended option of single-dose nevirapine during delivery, despite the higher cost of Option B [4]. Option B+ proposes that all HIV-infected pregnant women receive lifelong ART beginning at their first pregnancy, regardless of CD4 cell count [2]. This strategy may improve maternal health through reduced morbidity and mortality, and reduce overall MTCT, especially in settings with high fertility rates [2]. However, the cost implications of implementing Option B+ in resource-limited settings such as Ghana are uncertain and have not been thoroughly studied.

According to the Ghana AIDS Commission Sentinel Survey for 2013, an estimated 224,488 people were living with HIV/AIDS in Ghana, including 34,557 children (15% of total) [5]. Although the epidemic has stabilized with a seroprevalence of 1.3% in the general population and 1.9% among pregnant women, an estimated 2,407 new pediatric infections occurred in 2013, which accounts for 30% of all new infections [6]. Antiretroviral therapy was introduced to Ghana in 2003 and the program has been widely scaled up; as of the end of December 2012, there were a total of 165 ART sites in the country [7]. In 2001, the Ministry of Health of Ghana initiated a broad PMTCT program using single-dose nevirapine, and Option B was adopted in 2011, with 1,656 PMTCT sites established by December 2012. With more than 90% of pregnant women having access to antenatal care in Ghana, eliminating MTCT in the near future is within reach [5].

A key consideration when evaluating the benefits of continuous ART versus interrupted therapy is the time between successive pregnancies. Because Option B results in the cessation of ART following breastfeeding completion, a woman may not receive ART at the optimal starting point (i.e., before the end of the first trimester) of her next pregnancy. With Option B+, she remains continuously on ART, so there is no window during a future pregnancy when she is not receiving prophylactic therapy, assuming adherence to therapy.

In this study, we aim to evaluate the potential health benefits – to the mother and all future children – and cost-effectiveness of Option B+ versus Option B in Ghana, to help inform HIV therapy recommendations for pregnant women in resource-limited countries. In addition, we summarize primary data collected from two hospitals in Ghana, including the timing and number of pregnancies occurring over a woman’s lifetime. Prior studies have evaluated Option B+ in other settings, but to the best of our knowledge, no prior study has included multiple pregnancies in the economic evaluation of Option B+, which is needed to fully capture the impact of continuous therapy regimens in settings where many women have multiple children [8-11].

## Methods

We developed a state-transition model to compare the costs and benefits of two strategies (Option B versus Option B+) to prevent MTCT in Ghana. Transmission rates, life expectancies, and ART adherence rates were obtained from the Ghana Health Service [7,12]. Table 1 illustrates variables used in estimating HIV-related cost of care. Estimates for costs, utilities, disease progression rates, testing patterns by CD4 count, and development of ART resistance were obtained from published studies [7,13-20].

**Table 1:** **Costs considered in estimates of HIV-related care**

**Direct costs**	**Indirect costs – facility level**	**Indirect costs – program support**	**Costs not included in analysis**
Staff time caring for clients	Administrative staff time	Support from national ART program	Client time, transport, meals, and other client costs
Drugs to prevent & treat opportunistic infections	Supervision from regional level	Support from Ghana Health Service	Costs incurred by local communities
ARV drugs	Office equipment		Negative externalities
Medical consumables & supplies in clinic visits	Vehicles used for program administration		Technical assistance or administrative costs incurred by external donor agencies
Laboratory testing	Transportation costs for administration		
Medical equipment	Public utilities		
Physical infrastructure used for client care	Maintenance & repair		
	Staff training		
	Legal & auditing costs		

### Patient characteristics

The population under consideration is HIV-infected pregnant women in Ghana, who are pregnant with their first child. Data collection consisted of abstracting information about patient characteristics from paper medical charts at two government hospitals in Kumasi, Ghana (Table 2). These hospitals serve a range of women from Kumasi and its surrounding region, and their patients were considered representative of the overall population. All complete charts of HIV+ women receiving care at the two centers from January 2008 to June 2012 were reviewed. Based on the medical chart review, we obtained estimates of antenatal care access patterns, average baseline CD4 count at initiation of antenatal care, age of first pregnancy, lifetime number of children per woman, and time between successive pregnancies (Table 2). Maternal life expectancy was evaluated using medical charts and death records at each hospital. The study protocol was reviewed and approved by Institutional Review Boards at Yale School of Medicine and Kwame Nkrumah University of Science and Technology. No informed consent was obtained since patient information was anonymized and de-identified prior to analysis.

**Table 2:** **Characteristics of study population**

**Parameter**	**Count (% of total) or mean (** ±SD)*
Charts Reviewed	817
Suntreso Government Hospital	418 (51.2%)
Kumasi South Hospital	399 (48.8%)
Age of first pregnancy	22.78 years (±4.97 years)
Pregnant when diagnosed HIV-positive	223 (27.3%)
Baseline CD4 count	471 cells/mm^3^ (±299 cells/mm^3^)
CD4 < 350 cells/mm^3^ when pregnant and diagnosed	118 (52.9%)
Month of pregnancy when first accessing care (n = 92)	
1^st^ trimester	11 (12.0%)
Month 1	1 (1.1%)
Month 2	4 (4.4%)
Month 3	6 (6.5%)
2^nd^ trimester	37 (40.2%)
Month 4	11 (12.0%)
Month 5	14 (15.2%)
Month 6	12 (13.0%)
3^rd^ trimester	44 (47.8%)
Month 7	19 (20.7%)
Month 8	19 (20.7%)
Month 9	6 (6.5%)

*Percentages may not add to 100% due to rounding.

### Definition of options

Option B and Option B+ are the two most comprehensive options recommended for the prevention of MTCT by the WHO [21]. Under both options, an HIV+ pregnant woman with CD4 count <350 cells/mm^3^ is immediately treated with triple-ART that continues through life. Option B provides that women with CD4 > 350 cells/mm^3^ receive ART beginning at 14 weeks into gestation, throughout pregnancy, and after birth until breastfeeding ceases. Under Option B+, all women receive lifetime ART, even if CD4 count is >350 cells/mm^3^. Under both options, infants born to HIV+ women receive daily zidovudine (AZT) for the first 4–6 weeks of life [22].

### Model structure

**Figure 1:**
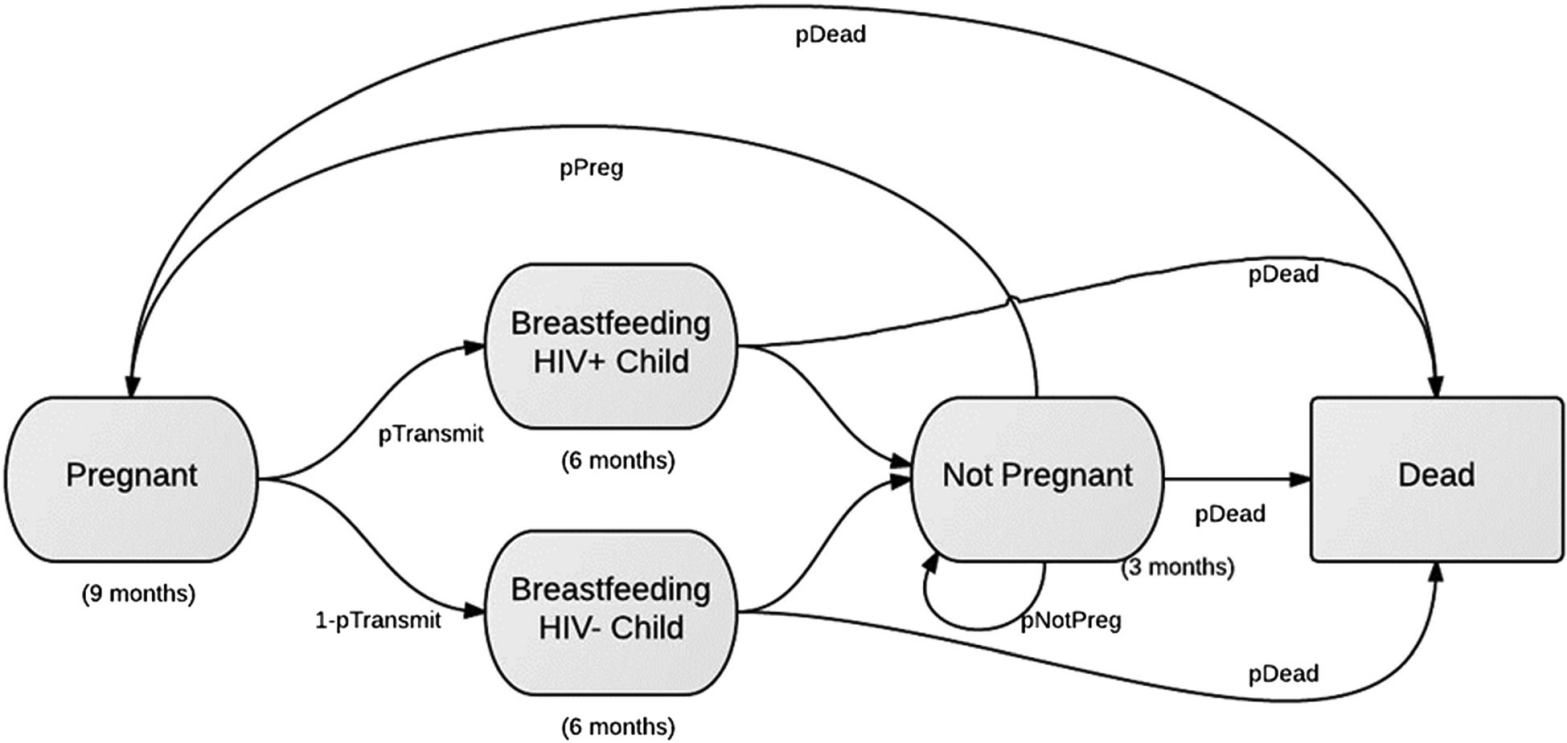
**State transition model overview.** A schematic diagram for the state transition model is given. Each oval represents a health state in which a woman can exist. She remains in a state for the period of time indicated underneath each oval. Each arrow represents a transition to the next state, which occurs with the probability indicated below each arrow.

We developed a state-transition model to calculate the average lifetime costs and health benefits associated with Option B+ or Option B (Figure 1). The model consists of several health states in which an HIV+ woman can exist, and transition probabilities that relate to the likelihood of moving to a different health state in the next time period. A woman remains in each state for a time unit of three months (a “cycle”), with the exception of the “Dead” state, in which a woman remains in this absorbing state.

To ensure appropriate lengths of pregnancy and breastfeeding, women are restricted to remain in the pregnancy state for nine months (3 cycles) and remain in either of the breastfeeding states for six months (2 cycles). The model initially assigns to each mother a starting age, CD4 cell count, and month of pregnancy, based on data obtained from chart review (Table 2). The model then tracks the mother’s CD4 count, pregnancy and antenatal care status, as well as the HIV status of her child following birth. Transitions between health states differed between treatment regimens, due to clinical differences in CD4 count decline and mortality. Unlike Markov models, which assume that all states are “memoryless” or ignore past states, our model allows for future transitions to depend on past events, such as number of previous pregnancies. By relaxing this Markov assumption, we can create a more clinically realistic model, whereby a woman’s past health states can impact her future states. Sensitivity analyses were conducted on all variables with ranges given in Table 3. The model was implemented in TreeAge Pro 2012. Additional file 1 model details are provided in the Technical Appendix.

**Table 3:** **Model parameters**

**Variable**	**Value**	**Range**	**Source**
***Demographic variables***			
Annual maternal mortality rates			UNICEF [20]
<12 months ART			
>350 cells/mm^3^	3.3%	-	
200-350 cells/mm^3^	3.9%	-	
<200 cells/mm^3^	11.1%	-	
>12 months ART			
>350 cells/mm^3^	1.0%	-	
200-350 cells/mm^3^	1.1%	-	
<200 cells/mm^3^	1.8%	-	
Number of children	2.34	1-5	Chart review
***Transition Probabilities***			
Access to care	82%	50-95%	Ghana Health Service [7]
Adherence to ART	90%	50-95%	Ghana Health Service [7]
Transmission during pregnancy			
No therapy/non-adherence	22%	15-30%	Ghana Health Service [12]
Option B	10%^Ϯ^	0-15%	Ghana Health Service [12]
Option B-Plus	1%	0-5%	Ghana Health Service [12]
Transmission during breastfeeding			
No therapy/non-adherence	10%	5-20%	Ghana Health Service [12]
Option B	1%	0-5%	Ghana Health Service [12]
Option B+	1%	0-5%	Ghana Health Service [12]
***Changes to CD4 Count***			
No therapy (every 3 months)	−12.75 cells/mm^3^	5-20 cells/mm^3^	Holmes 2006 [15]
Initiate therapy	+153 cells/mm^3^	100-400 cells/mm^3^	Deeks 1999 [16]
Continue therapy (every 3 months)			
Previously interrupted therapy	−0.06365 × [Current CD4]	-	Ickovics et al. 2001 [14]
Continuous therapy	- 0.0099853 × [Current CD4]	-	Ickovics et al. 2001 [14]
***Costs in USD [GHS]***			
Annual Cost of HIV Care			
First-Line ART	385.45 [743.91]	191-580	WHO 2011 [17]; Rosen, J., and F. Asante. 2010 [18]
Second-Line ART	848.33 [1,637.28]	-	WHO 2011 [17]; Rosen, J., and F. Asante. 2010 [18]
Lifetime Cost of Care for HIV+ Child (including ART costs)	10,665.49 [20,584.40]	5,181-15,544	WHO 2011 [17]; Rosen, J., and F. Asante. 2010 [18]
***Quality-of-life Factor***			
HIV+ adult (quality of life)	0.8	0.50-1.0	Tengs T.O., Lin T.H. 2002 [19]
HIV+ child (lifetime QALYs)	20.2^ϮϮ^	10-30	UN Impact of AIDS 2004 [32]
HIV- child (lifetime QALYs)	62.7	50-70	UN Impact of AIDS 2004 [32]
Discount rate	0.03	0.00-0.05	Weinstein et al. 1996 [23]

^Ϯ^Probability of transmission during pregnancy/delivery while on Option B was determined by applying the distribution of when women accessed antenatal care (found through chart review) with ideal conditions of Option B (beginning therapy at the beginning of the second trimester) indicated by the Ghana Health Service and WHO.

^ϮϮ^Lifetime QALYs for an HIV-positive child assume a life expectancy at birth of 47.1 years [32] with a yearly utility of 0.82 and a discount rate of 0.03.

### Key model parameters

#### Fertility rates

**Figure 2:**
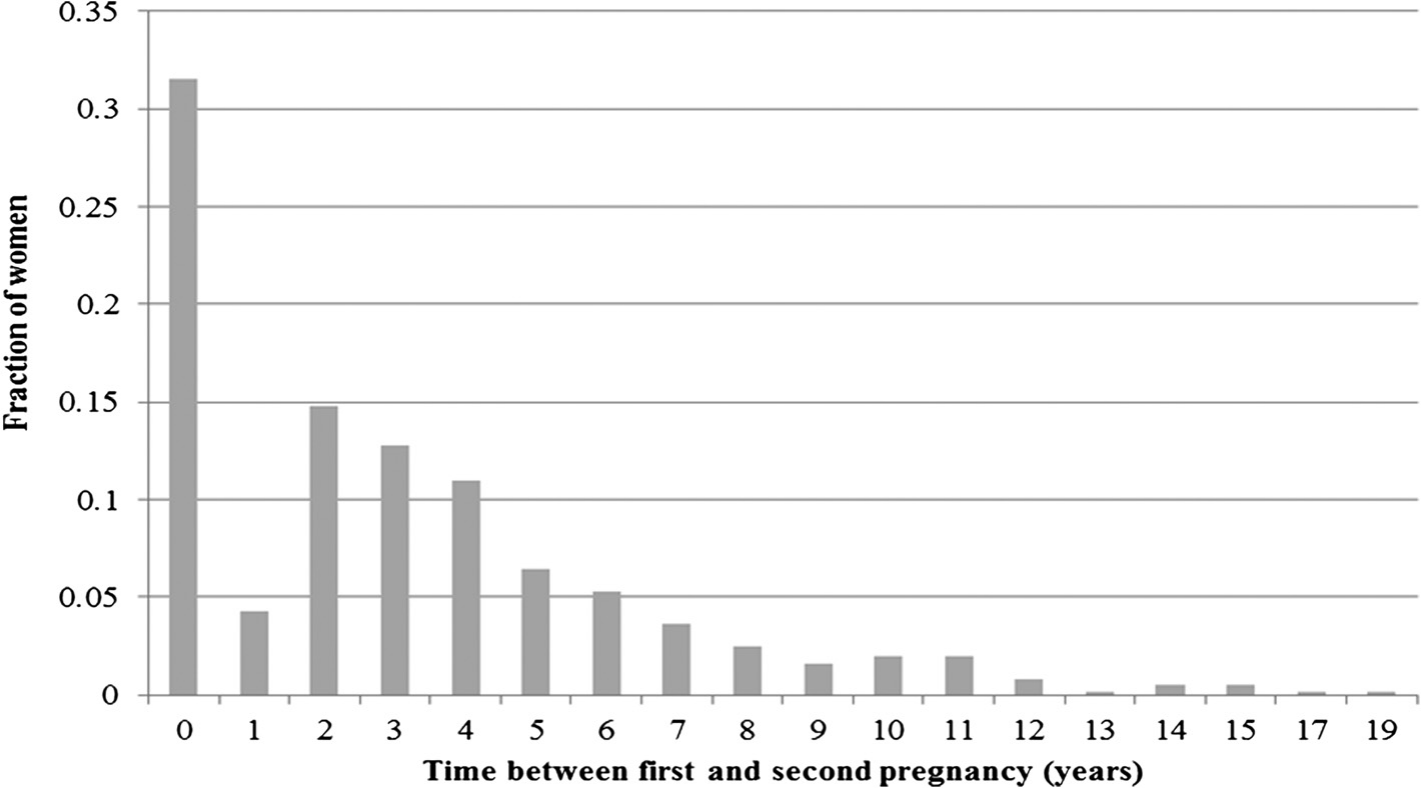
**Probability distribution of time between 1** ^**st**^ **and 2** ^**nd**^ **pregnancy.** The fraction of women (n = 817) who wait a given number of years between their first and second pregnancy is shown, with the number of years indicated on the x-axis and the proportion of women indicated on the y-axis. “0 years” indicates that the mother had only one child.

Based on medical chart review, a probability hazard function was fit to estimate the rate of future pregnancies following the first pregnancy (Figure 2). Each pregnancy recorded from the chart review was considered an event and the age at which these events occurred was included in the model. This hazard model then produced the probability of a subsequent pregnancy occurring at each age in a woman’s lifetime. The model was developed in SAS 9.3.

#### Access to antenatal care

In Ghana a pregnant woman initiates antenatal care at a health facility during the first trimester of pregnancy; there are at least four antenatal care visits for an uncomplicated pregnancy [21]. The routine services provided at antenatal visits include assessing for pregnancy complications (e.g., anemia, hypertension, and bleeding), nutritional advice, immunization, HIV testing and counselling, monitoring of pregnancy progress, and assessment of maternal and fetal well-being. For HIV-infected pregnant women, monitoring of ART side effects and compliance to treatment are assessed at each visit. The overall fraction of pregnant women in Ghana who receive antenatal care at some point in their pregnancy was determined from a national survey [7]. We determined the proportions of pregnant women accessing antenatal care, by month of pregnancy, from medical chart review.

#### ART initiation and adherence

Estimates of ART adherence and the probability of mother-to-child transmission during pregnancy, delivery, or while breastfeeding were obtained from annual reports issued by the Ghana Health Service (Table 3) [7,12]. Under Option B, a woman who does not qualify for lifetime therapy at her first pregnancy (by having a CD4 cell count >350 cells/mm^3^) can initiate ART once when her CD4 cell count decreases below 350 cells/mm^3^. Women who qualify are provided ART at the first possible event – either pregnancy or CD4 below the 350 cells/mm^3^ threshold [8]. Our model tracks CD4 cell count throughout a woman’s lifetime and accounts for ART initiation once this threshold is reached.

#### Mother-to-child transmission

Estimates of transmission rates dropping to 1% among women receiving Option B assume ideal conditions (i.e., a pregnant woman receives therapy from the beginning of her first trimester through six months of breastfeeding) [12]. However, these rates are not achievable if antenatal care is initiated beyond the first trimester of pregnancy. To estimate a more realistic transmission rate for Option B, we multiplied the percentage of women accessing antenatal care during each month of pregnancy by a scaled transmission rate. This scaled MTCT rate increased from 1.0% if a woman accessed antenatal care during the first three months of pregnancy, up to 20.3% if antenatal care began in the ninth month, generating an overall transmission rate during pregnancy/delivery of 10.2% for all pregnancies under Option B. Under Option B+, this rate applied for only the first pregnancy; subsequent pregnancies assumed a transmission rate of 1.0%. Further details can be found in Additional file 1: Table S2 of the Technical Appendix. Transmission rates were examined under robust sensitivity analyses in case adherence rates increase or decrease.

#### Additional model parameters

Additional values not available through chart review were acquired from previously published studies. All costs are reported in US Dollars (1 USD = 1.93 Ghana Cedi, abbreviated as GHS) and incorporate all components of HIV/AIDS care following diagnosis, including ART, medical personnel wages, and CD4 count and viral load testing. In particular, we assumed that the annual cost of HIV/AIDS care with first-line ART is $385 or 744 GHS (3-month cost of $96 or 186 GHS), but we consider variations of this assumption in sensitivity analysis (Table 3) [17,18].

Changes in CD4 cell count were modeled from previously published works and the rate of CD4 change was determined by a woman’s ART utilization and whether ART is interrupted or continuous [14,15]. Women incurred a CD4 cell count increase of 153 cells/mm^3^ at the initiation of therapy [16].

Adjustments for quality-of-life while living with HIV were considered as a yearly adjustment for adults and a lifetime adjustment for children. Adult women living with HIV were assumed to have 0.8 times the quality-of-life of otherwise healthy women [19]. Children born HIV+ were attributed this same 0.8 quality-of-life adjustment, applied across life expectancy and discounted at an annual rate of 3% [23].

Mortality rates were obtained from a Business Leadership Council/UNICEF report and considered a woman’s CD4 count, as well as the length of time she has been receiving therapy [20].

### Model outcomes

Primary outcomes of the model were costs and quality-adjusted life years (QALYs) for each therapy option. All costs and QALYs were discounted to the present using a 3% annual rate [23]. Costs and QALYs of Option B and Option B+ were then compared using an incremental cost-effectiveness ratio (ICER):

$$ ICER=\frac{Cos{t}_{OptionB+}-Cos{t}_{OptionB}}{QALY{s}_{OptionB+}- QALY{s}_{OptionB}} $$

ICER values were measured in cost per QALY gained, and then compared to benchmarks established by the WHO Commission on Macroeconomics for Health, which state that “cost-effective” health interventions are those with an ICER less than three times gross domestic product (GDP) per capita, and “very cost-effective” interventions are those with an ICER less than GDP per capita [24]. GDP per capita in Ghana was $3,300 (6,369 GHS) in 2012 [25].

## Results

### Characteristics of study population

A total of 817 medical charts were reviewed – 418 at Suntreso Government Hospital and 399 at Kumasi South Hospital (Table 2). The average age of first pregnancy was 22.78 years (SD: 4.97 years) and the average lifetime number of children per woman was 2.34 (SD: 1.27 children). Among women whose timing of antenatal care access was known, 12% accessed care in their first trimester, 40% in their second trimester, and 48% in their third trimester.

### Pregnancy frequency

The time to a second pregnancy following the first pregnancy was taken directly from chart review data (Figure 2). Approximately 32% of women in our sample did not have a second pregnancy, while 4% became pregnant within one year following their first pregnancy and 19% became pregnant within two years. The average time between the first and second pregnancies was 4.56 years (SD: 3.05 years).

### Reduction in mother-to-child transmission

Approximately 10,800 births occur among HIV+ women in Ghana each year [26]. Further, based on chart review, 68% of those births (7,344 births) are not the mother’s first child. Under Option B, our model projects that the average rate of MTCT is 10.2% during pregnancy or delivery. The Ghana Health Services estimates an additional MTCT rate of 1% during breastfeeding [12]. If all pregnant women in Ghana received Option B, our model projects that 814 children would acquire HIV from their mothers each year. Under Option B+, the HIV transmission rate is 1% through pregnancy or delivery, and also 1% during breastfeeding. If all women in Ghana were instead offered Option B+ and had perfect adherence, a projected 146 infections would occur in children each year. Offering Option B+ in lieu of Option B could thus theoretically prevent up to 668 HIV infections among newborn babies in Ghana each year.

### Cost-effectiveness analysis

Our model estimates that maternal life expectancy is 16.1 years (discounted) with Option B+, compared to 16.0 years with Option B, a gain of 0.1 years on average because of fewer treatment interruptions (Table 4). Additionally, Option B+ increases average health benefits per child from 63.6 QALYs to 66.8 QALYs, due to reduced MTCT in subsequent pregnancies. Aggregating the QALYs attributed to the mother as well as her current child and all future children, Option B+ yields 180.2 total QALYs, a substantial gain over 172.1 QALYs expected under Option B. However, Option B+ is also more costly, requiring lifetime total costs (discounted) of $12,624 compared to only $6,254 with Option B. The incremental cost-effectiveness is thus $785 per QALY gained, or $618 per life-year gained (if quality-of-life weights are ignored).

**Table 4:** **Baseline results of primary outcomes**

**Treatment strategy**	Lifetime cost (GHS)*	Lifetime cost (USD)*	Life-years*	QALYs* ^**Ϯ**^	**ICER (USD)** ^**Ϯ**^
***Option B***	12,071	6,254	186.4	172.1	
Mother	2,617	1,356	16.0	13.1	
Children	9,453	4,898	178.5	159.0	
Per Child	3,781	1,959	71.4	63.6	
***Option B+***	24,364	12,624	196.7	180.2	785
Mother	18,821	9,752	16.1	13.2	
Children	5,544	2,872	178.4	167.0	
Per Child	2,217	1,149	71.4	66.8	

*All costs, life-years, and QALYs are discounted by 3% annual rate.

^Ϯ^QALY = Quality-Adjusted Life-Year; ICER = Incremental Cost-Effectiveness Ratio.

All QALYs are calculated using an adjustment of 0.82 utility per year when living with HIV and an annual discount rate of 0.03.

The ICER value is calculated as follows: $ICER=\frac{Cos{t}_{OptionB+}-Cos{t}_{OptionB}}{QALY{s}_{OptionB+}- QALY{s}_{OptionB}}.$

### Sensitivity analyses

A detailed sensitivity analysis was performed on all model parameters, to test for robustness and identify key parameters impacting cost-effectiveness results. Of all variables examined, cost-effectiveness was most sensitive to the cost of antiretroviral therapy for the mother on Option B+. However, even when the annual cost of HIV care is increased from $385 to $580, the ICER increased to only $1,358 per QALY gained, still below the Ghanaian GDP per capita of $3,300.

**Figure 3:**
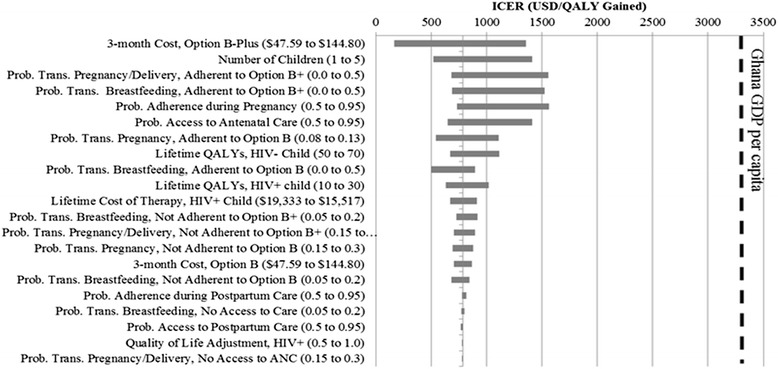
**Sensitivity analysis of model variables.** This tornado diagram represents the sensitivity of different variables included in the model. Each variable is listed, along with the associated incremental cost-effectiveness ratio (ICER). The horizontal width of each bar represents the change in cost-effectiveness of Option B+ versus Option B ($/QALY gained) as each model parameter is varied over the range given in parentheses. Variables are listed in descending sensitivity; those whose ICER values change most significantly are listed first. For reference, a vertical line indicating the Ghanaian GDP per capita is included on the graph, which demonstrates that Option B+ is a cost-effective alternative, even across a wide range of sensitivity analyses. The base case ICER value is $785/QALY gained.

**Figure 4:**
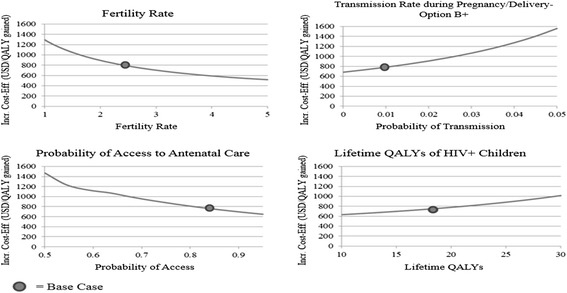
**One-way sensitivity analyses.** Each graph indicates the change in the incremental cost-effectiveness ratio (ICER) between Option B and Option B+ when a single variable’s value is changed. The “base case” scenario is indicated with a circle. A downward-sloping line indicates Option B+ is becoming more cost-effective as the variable’s value is increased, while an upward-sloping line indicates Option B+ is becoming less cost-effective as the variable’s value is increased. The curve of each line indicates the specific rate at which the ICER changes as the variable’s value is altered.

A tornado diagram (Figure 3) shows that the cost-effectiveness of Option B+ was also sensitive to life expectancies, disease transmission probabilities, access to antenatal care, and fertility rates. One-way sensitivity analyses of several variables display each variable’s relationship to the ICER value (Figure 4). In general, as the probability of accessing antenatal care changes from 50% to 95%, Option B+ becomes more cost-effective because future children are more likely to avoid HIV infection. Conversely, as maternal life expectancy on Option B increases, the cost-effectiveness of Option B+ worsens because the marginal gain in QALYs resulting from Option B+ diminishes.

Finally, the cost-effectiveness of Option B+ improves as the fertility rate increases; with five children per mother, the ICER drops to $500 per QALY gained. Of note, if we consider only one child per mother – the assumption imposed by most MTCT modeling studies – we obtain an ICER of $1,300 per QALY gained, a value 75% higher than our initial estimate, highlighting the importance of including future pregnancies in cost-effectiveness estimates.

## Discussion

Our findings suggest that offering the Option B+ therapy regimen to HIV+ pregnant women in Ghana improves both maternal and child outcomes, and that the additional cost of such a program is likely warranted given its favorable cost-effectiveness. Option B+ provides continuous therapy to women during their index pregnancy and all subsequent pregnancies. This represents a significant improvement over Option B, whereby treatment interruption often delays ART receipt until the second or third trimester of later pregnancies. Our modeling framework was novel in its ability to account for multiple pregnancies, and we find that ignoring HIV transmission to future children may underestimate the potential benefits and cost-effectiveness of Option B+.

Our study has important implications for helping policymakers allocate limited HIV resources more effectively. Option B+ costs approximately $785 per QALY gained, compared to Option B, similar to other established HIV prevention methods in Ghana, including voluntary HIV counseling and blood donation screening [4,27]. This value is in line with other HIV interventions shown to be cost-effective in low- and middle-income countries, such as male circumcision, HIV screening and counseling, and school-based education [28-30]. Our results are generally consistent with other model-based studies estimating the cost-effectiveness of Option B+ in Nigeria, Malawi, and Zimbabwe [8,9,11]. These previous analyses demonstrated the economic merit of Option B+, and our analysis extends these findings by incorporating the possibility of multiple pregnancies over a woman’s lifetime, and including primary data collection from antenatal clinics. Based on guidelines proposed by the WHO Commission on Macroeconomics in Health, Option B+ is considered very cost-effective compared to Option B [24]. These results are robust to wide variations in parameter values, suggesting that Option B+ is likely cost-effective in settings with similar resources and epidemiologic characteristics to Ghana. A nationwide roll-out of Option B+ would of course require investment in health systems to ensure ART adherence and provide early antenatal care to pregnant women. However, such investment would likely further improve maternal and child health outcomes for other diseases such as malaria, tuberculosis, and childhood malnutrition.

Our modeling study has several limitations. First, because Option B+ is a newly recommended strategy, additional data on its efficacy and impact on maternal life expectancy are evolving, although we have tested variations in these assumptions in sensitivity analysis. Second, the model does not explicitly consider horizontal HIV transmission; however, we believe this is a reasonable assumption because an HIV+ pregnant woman is likely to have a regular partner who is also HIV+ [31]. We did not consider variations in the duration or exclusivity of breastfeeding practices, although variability in behavior will inevitably exist. We estimated the expected costs and QALYs associated with Option B and Option B+, but the model simplifies complex clinical outcomes, such as viral load, development of opportunistic infections, averted costs, HIV+ women initiating ART prior to first pregnancy, or variability in HIV progression among children.

## Conclusions

In resource-limited settings such as Ghana, systematically comparing the potential health benefits and costs of competing HIV programs can illuminate where additional investment should be prioritized. Option B+ provides considerable health benefits to HIV+ women and their children – especially in settings where women have multiple pregnancies – and represents good value. With nationwide implementation of Option B+, we estimate that up to 668 newborn children would be prevented from acquiring HIV in Ghana every year, and preventing these infections now is a key step towards reducing the burden of HIV in the future.

## Additional file

Additional file 1:
**Table S1.** Average time between pregnancies. **Table S2.** Access to antenatal care, by month of pregnancy. **Table S3.** Annual Mortality Rate of HIV+ Women. **Table S4.** Chart Review – Full Results. **Figure S1.** MTCT probability by month of antenatal care access. **Figure S2.** Schematic model diagram comparing Option B with Option B Plus.

## Abbreviations

MTCT: Mother-to-child transmission
ART: Antiretroviral therapy
QALYs: Quality adjusted life-years
HIV: Human immunodeficiency virus
WHO: World Health Organization
AZT: Zidovudine
SDNVP: Single dose nevirapine
PMTCT: Prevention of mother-to-child transmission
UNICEF: United Nations Children’s Fund
ICER: Incremental cost-effectiveness ratio
GDP: Gross domestic product

## References

[1] UNAIDS. World AIDS Day Report. 2012. [http://www.unaids.org/en/media/unaids/contentassets/documents/epidemiology/2012/gr2012/JC2434_WorldAIDSday_results_en.pdf]

[2] UNAIDS. Eliminating new HIV infections among children. [http://www.unaids.org/en/targetsandcommitments/eliminatingnewhivinfectionamongchildren/]

[3] WHO. Global Monitoring Framework and Strategy for the Global Plan towards the elimination of new HIV infections among children by 2015 and keeping their mothers alive (EMTCT). 2012. [http://apps.who.int/iris/bitstream/10665/75341/1/9789241504270_eng.pdf]

[4] Baiden F, Baiden R, Williams J, Akweongo P, Clerk C, Debpuur C (2005). Review of antenatal-linked voluntary counseling and HIV testing in Sub-Saharan Africa: lessons and options for Ghana. Ghana Me J.

[5] UNICEF. At a Glance: Ghana - Statistics. 2007. [http://www.unicef.org/infobycountry/ghana_statistics.html]

[6] Ghana AIDS Commission. Ghana - Country AIDS Progress Report. Reporting Period: January 2010-December 2011. UNAIDS. 2012. [http://www.unaids.org/sites/default/files/en/dataanalysis/knowyourresponse/countryprogressreports/2012countries/ce_GH_Narrative_Report%5B1%5D.pdf]

[7] Ghana Health Service (2011). PMTCT annual report.

[8] Shah M, Johns B, Abimiku A, Walker DG (2011). Cost-effectiveness of new WHO recommendations for prevention of mother-to-child transmission of HIV in a resource-limited setting. AIDS.

[9] Ciaranello AL, Perez F, Engelsmann B, Walensky RP, Mushavi A, Rusibamayila A (2013). Cost-effectiveness of World Health Organization 2010 guidelines for prevention of mother-to-child HIV transmission in Zimbabwe. Clin Infect Dis.

[10] Kuznik A, Lamorde M, Hermans S, Castelnuovo B, Auerbach B, Semeere A (2012). Evaluating the cost-effectiveness of combination antiretroviral therapy for the prevention of mother-to-child transmission of HIV in Uganda. Bull World Health Organ.

[11] Fasawe O, Avila C, Shaffer N, Schouten E, Chimbwandira F, Hoos D (2013). Cost-effectiveness analysis of Option B+ for HIV prevention and treatment of mothers and children in Malawi. PLoS One.

[12] National AID, Program C (2010). PMTCT training package for health care providers participant manual.

[13] Sterne JA, May M, Costagliola D, de Wolf F, Phillips AN, When To Start Consortium (2009). Timing of initiation of antiretroviral therapy in AIDS-free HIV-1-infected patients: a collaborative analysis of 18 HIV cohort studies. Lancet.

[14] Ickovics JR, Hamburger ME, Vlahov D, Schoenbaum EE, Schuman P, Boland RJ (2001). Mortality, CD4 cell count decline, and depressive symptoms among HIV-seropositive women: longitudinal analysis from the HIV epidemiology research study. JAMA.

[15] Holmes CB, Wood R, Badri M, Zilber S, Wang B, Maartens G (2006). CD4 decline and incidence of opportunistic infections in Cape Town, South Africa: implications for prophylaxis and treatment. JAIDS.

[16] Deeks SG, Grant RM (1999). Sustained CD4 responses after virological failure of protease inhibitor-containing therapy. Antivir Ther.

[17] WHO. Global Price Reporting Mechanism Report AIDS Medicines and Diagnostics Service. WHO; 2011. (http://www.who.int/hiv/amds/gprm/en/).

[18] Rosen J, Asante F (2010). Cost of HIV & AIDS adult and pediatric clinical care and treatment in Ghana.

[19] Tengs TO, Lin TH (2002). A meta-analysis of utility estimates for HIV/AIDS. Med Decis Making.

[20] UNICEF (2012). Business leadership council. A business case for options B and B+ to eliminate mother to child transmission of HIV by 2015.

[21] WHO. Antiretroviral Drugs for Treating Pregnant Women and Preventing HIV Infection in Infants: Towards Universal Access - Recommendations for a public health approach. World Health Organization; 2010. [http://www.who.int/hiv/pub/guidelines/pmtctguidelines3.pdf] 26180894

[22] UNICEF (2012). Options B and B+: Key considerations for countries to implement an equity-focused approach.

[23] Weinstein MC, Siegel JE, Gold MR, Kamlet MS, Russell LB (1996). Recommendations of the panel on cost-effectiveness in health and medicine. JAMA.

[24] WHO Commission on Macroeconomics and Health (2001). Macroeconomics and health: investing in health for economic development.

[25] USA Central Intelligence Agency: World Factbook - Ghana. [https://www.cia.gov/library/publications/the-world-factbook/geos/gh.html]

[26] United Nations (2012). Together We will End AIDS. Joint united nations programme on HIV/AIDS.

[27] van Hulst M, Sagoe KW, Vermande JE, van der Schaaf IP, van der Tuuk Adriani WP, Torpey K (2008). Cost-effectiveness of HIV screening of blood donations in Accra (Ghana). Value Health.

[28] Kahn JG, Marseille E, Auvert B (2006). Cost-effectiveness of male circumcision for HIV prevention in a South African setting. PLoS Med.

[29] Long E, Stavert RR (2013). Portfolios of biomedical HIV interventions in south Africa: a cost-effectiveness analysis. J Gen Intern Med.

[30] Hogan DR, Baltussen R, Hayashi C, Lauer JA, Salomon JA (2005). Cost effectiveness analysis of strategies to combat HIV/AIDS in developing countries. BMJ.

[31] Achana FS, Debpuur C, Akweongo P, Cleland J (2010). Postpartum abstinence and risk of HIV among young mothers in the Kassena-Nankana District of Northern Ghana. Cult Health Sex.

[32] United Nations: The Impact of AIDS. United Nations, Department of Economic and Social Affairs PD; 2004. [http://www.un.org/esa/population/publications/AIDSimpact/1CoverNotePrefaceContents.pdf]

