# Supplementary material for: Cost effectiveness of option B plus for prevention of mother-to-child transmission of HIV in resource-limited countries: evidence from Kumasi, Ghana
Source: BMC Infect Dis. 2015 Mar 18;15:130. doi: 10.1186/s12879-015-0859-2 (PMC4374181; doi:10.1186/s12879-015-0859-2)
Supplement: Additional file 1: — Table S1. Average time between pregnancies. Table S2. Access to antenatal care, by month of pregnancy. Table S3. Annual Mortality Rate of HIV+ Women. Table S4. Chart Review – Full Results. Figure S1. MTCT probability by month of antenatal care access. Figure S2. Schematic model diagram comparing Option B with Option B Plus. [file 12879_2015_859_MOESM1_ESM.doc]

# A Case for Adopting Option B Plus for Prevention of Mother-to-Child Transmission of HIV in Resource-Limited Countries: Evidence from Kumasi, Ghana

**APPENDIX**

Adam VanDeusen, Elijah Paintsil, Thomas Agyarko-Poku, Elisa F. Long

**Model Overview**

The model used in this analysis was a decision-analytic, semi-Markov model (Figure A2). The model consists of several states in which a woman can exist, with specific transition probabilities associated with future states following her current state. A woman remains in each state for a time unit of 3 months, with the exception of the “Dead” state, in which a woman completes model transitions and remains in this absorbing state. To ensure appropriate lengths of pregnancy and breastfeeding, women are restricted to remain in the pregnancy state for 9 months (3 cycles) and remain in either of the breastfeeding states for 6 months (2 cycles). An overview of the model’s states and transitions can be found in Figure 1 of the paper.

**Model Parameter Calculations**

*Pregnancy Rate*

Through detailed medical chart review of 817 HIV+ women, the average number of children born to each HIV+ woman was found to be 2.34 children (SD 1.27). The average time between pregnancies is shown in Table A1. To calculate the fertility rate, all ages at which pregnancies occurred were included in a survival analysis conducted in SAS 9.3, with a pregnancy indicating an event of interest. This survival analysis yielded the probability of pregnancy occurring at each age in a woman’s life. We assumed a constant pregnancy rate within each year. We used annual pregnancy probabilities to compute the probability of pregnancy in each 3-month period.

**Table A1. Average time between pregnancies.**

| **Pregnancy Number** | **Time Between Pregnancies (Years) Mean ± SD** |
| --- | --- |
| 1-2 | 4.56 ± 3.05 |
| 2-3 | 4.05 ± 2.71 |
| 3-4 | 3.67 ± 2.29 |
| 4-5 | 3.30 ± 1.96 |
| 5-6 | 3.73 ± 1.42 |

*Age of First Pregnancy*

Age of first pregnancy was calculated by considering ages of first pregnancy among all charts reviewed. In total, 610 charts included this value and the average age of first pregnancy was 22.78 years (SD 4.97), with a range of 11-40 years. In our model, we used this 23 years as a starting age for all women.

*Transmission Rate during Pregnancy/Delivery*

Without therapy, the rate of HIV transmission from mother-to-child during pregnancy and delivery is 20-25% [1]. Ideally, women should access antenatal care (ANC) during their first trimester of pregnancy and begin ART before the start of their second trimester (month 4). If this occurs, women receive a full dose of ART and are able to reduce the probability of transmission to approximately 1%. However, the women in our population did not access ANC during their first trimester – almost 50% accessed ANC during their third trimester, thereby attenuating the effects of ART on transmission. Assuming that the likelihood of transmission increases linearly with each subsequent month following month 4 if ART is not utilized (Figure A1), we calculated an overall transmission rate for Option B during pregnancy and delivery. The percentage of women first accessing ANC in each month is derived through chart review. Because women are continuously on therapy under Option B+, this transmission rate is not dependent on the month of ANC access and is always considered to be 1% [1]. If women did not access care or were not adherent to care, they incurred the untreated transmission rate of 22% [1].

**Figure A1. MTCT probability by month of antenatal care access**


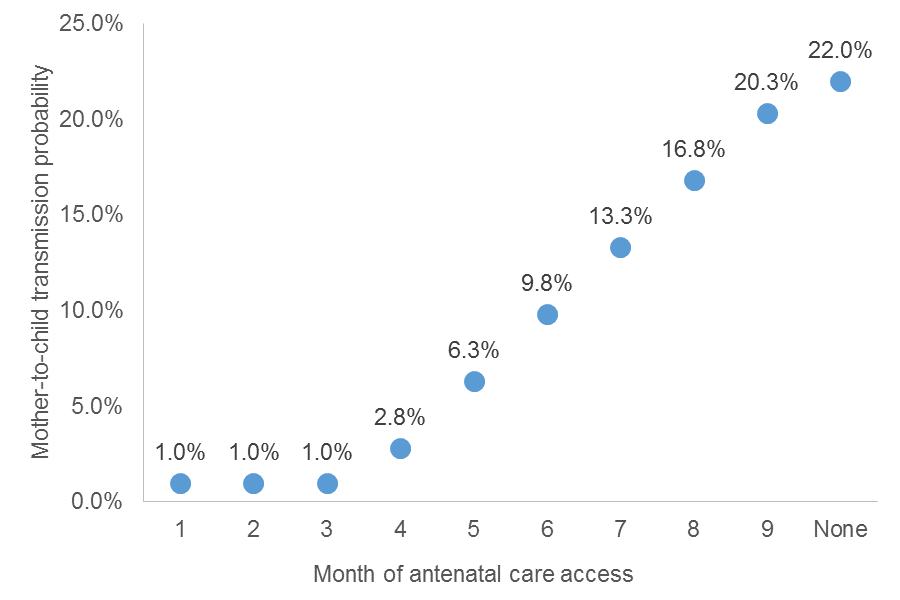


**Table A2**. Access to antenatal care, by month of pregnancy

| Month: | 1 | 2 | 3 | 4 | 5 | 6 | 7 | 8 | 9 |
| --- | --- | --- | --- | --- | --- | --- | --- | --- | --- |
| % of women first accessing antenatal care (p_month_) | 1.1 | 4.3 | 6.5 | 12.0 | 15.2 | 13.0 | 20.7 | 20.7 | 6.5 |
| MTCT probability (rate_month_) | 1.0 | 1.0 | 1.0 | 2.8 | 6.3 | 9.8 | 13.3 | 16.8 | 20.3 |


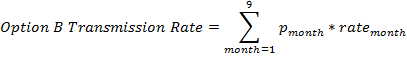


*Transmission Rate during Breastfeeding*

Without therapy, the rate of HIV transmission from mother-to-child during breastfeeding is 10-15%. Our model considers 10% as the baseline rate of transmission for this parameter (though sensitivity analyses incorporate ranges from 0-15%). When women can access therapy and are adherent to it, the transmission rate is reduced to approximately 1%. Because women following Option B or Option B+ would have relatively similar timing of accessing therapy during breastfeeding (due to this period immediately following delivery), this rate was not considered different when comparing the options.

*Access to Antenatal Care & Adherence to Therapy*

Access to antenatal care was determined from a report from the Ghana Health Service. This report included results of a national survey of Ghanaian citizens on various aspects of healthcare utilization, including access to healthcare services. Overall, 82.17% of individuals reported having access to hospital-based care, based on a binary question [1].

Adherence to therapy was also determined from the same Ghana Health Service report. Individuals reported being fully adherent to prescribed therapies in 80.0% of cases. More objective measures, such as records of prescription pick-up from pharmacies or pill counting during clinical visits were not logistically or administratively feasible. The rate used in this analysis is subject to information bias, which was accounted for during sensitivity analyses.

*Maternal Death Rate*

Death rates were determined based on a woman’s CD4 count and time spent on antiretroviral therapy, as indicated in Table A3. In the model, these annual rates were converted to quarterly rates to align with the model format. Rates were derived from a Business Leadership Council/UNICEF report [2].

**Table A3**. Annual Mortality Rate of HIV+ Women

|  | On ART <12 months | On ART >12 months |
| --- | --- | --- |
| CD4 >350 cells/mm^3^ | 3.3% | 1.0% |
| CD4 200-350 cells/mm^3^ | 3.9% | 1.1% |
| CD4 <200 cells/mm^3^ | 11.1% | 1.8% |

*Child Life Expectancy*

Life expectancy of children was determined using values from literature. Life expectancy of an HIV+ child is 47.1 years and life expectancy of an HIV- child is 62.7 years [3]. These values are adjusted using an annual discount rate and the life expectancy of an HIV+ child is also adjusted for reduced quality-of-life.

*Cost of HIV-Related Care*

Costs of HIV-related care were estimated using a 2010 Ghana Health Service report on costs and treatment of HIV in Ghana. Cost estimates included the components outlined in Table 1 in the manuscript. The cost of HIV care for an adult on first-line therapy was 189.5 Ghana Cedi (GHS) per 3-months, while the cost was 409.3 GHS per 3-months for an adult on second-line therapy. The lifetime cost of an HIV-positive child’s care was 20,584.40 GHS.

*Quality of Life Adjustment*

All mothers in the model incurred a quality-of-life adjustment of 0.8, a standard value for adults living with HIV. This adjustment was applied to all life-years to convert to QALYs. In other words, an HIV+ woman who lived for one year accrued 0.8 QALYs. Discount rates were also applied to all QALYs gained.

All HIV-positive children incurred a quality of life adjustment of 0.82. This was applied to each year of life expectancy to determine lifetime QALYs per child. Discount rates were also applied to lifetime QALYs for children.

The life-years of HIV-negative children were not quality adjusted, indicating 1 QALY gained for each year the children were expected to live. Discount rates were applied to lifetime QALYs for HIV- children.

*Discount Rate*

An annual discount rate of 3% was used for all costs and QALYs in the model to ensure comparisons between Options B and B+ were being made across the appropriate time. Due to the model’s 3-month cycle length, each cost and QALY in the model was adjusted by the following factor, where *t* represents the number of periods following model initiation:


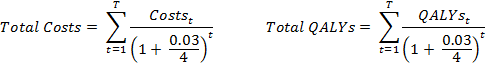


**Model Transitions**

As demonstrated in the state-transition model (Figure 1 in main text), women can “exist” in five main states and transition between these states with probabilities that are dependent on transmissions, therapy option (B or B+), age, and other factors. In this section, we will discuss how women transition between states in the model. The model was developed in TreeAge Pro (2012 edition).

*Pregnant State to Breastfeeding States*

The model begins with all women entering the pregnant state (Figure A2). A tunnel state restricts women to remain in this state for 9 months. Under Option B, women receive a boost in CD4 cell count associated with therapy initiation. Under Option B+, women receive this boost in CD4 cell count only during their first pregnancy. Following pregnancy, women enter one of the breastfeeding states, based on whether or not they transmit HIV to their child during pregnancy/delivery. Prior to transmission probabilities being assessed, women are first evaluated for access to care and adherence to therapy. If women are both able to access to care and adherent to therapy, they have a reduced transmission of transmitting HIV. If a woman does not access care or is not adherent to therapy, her likelihood of transmission remains as though she did not receive therapy.

*Breastfeeding States to Not Pregnant States*

If a mother transmits HIV to her child during pregnancy or delivery, she enters the “Breastfeeding HIV+ Child” state. She remains in this state for 6 months and then moves to being “Not Pregnant.” Costs are incurred only if the woman is able to access postpartum care.

If a mother does not transmit HIV to her child during pregnancy/delivery, she enters the “Breastfeeding HIV- Child” state. She remains in this state for 6 months before moving to the “Not Pregnant” state. While in the “Breastfeeding HIV- Child” state, a woman may still transmit HIV to her child, again based on access to postnatal care and adherence to antiretroviral therapy.

*Not Pregnant State to Pregnant State*

Once a woman enters the “Not Pregnant” state, she is evaluated every 3 months to determine if she remains not pregnant, becomes pregnant again, or dies. Under Option B, women are continuously tracked using a tracker variable, to evaluate their CD4 cell count. If CD4 count falls below 350 cells/mm^3^, women receive therapy while not pregnant; otherwise they remain off therapy while not pregnant. A “Not Pregnant” woman transitions to a “Pregnant” state based on her age-specific fertility rate (Table A1).

*All States to Dead State*

In all states, a woman has a probability of dying associated with CD4 count (>350 cells/mm^3^, 200-350 cells/mm^3^, and <200 cells/mm^3^) and length of time she has been on antiretroviral therapy (≥12 months or < 12 months).

**Table A4. Chart Review – Full Results**

| **Patient Characteristic** | **N (% of total)** |
| --- | --- |
| Location (n=817) |  |
| Suntreso Government Hospital | 418 (51.2%) |
| Kumasi South Hospital | 399 (48.8%) |
| Pregnancy Status (n=588) |  |
| Pregnant | 221 (37.6%) |
| Not pregnant | 367 (62.4%) |
| First Access of Antenatal Care (n=92) |  |
| First trimester | 11 (12.0%) |
| Second trimester | 37 (43.5%) |
| Third trimester | 44 (47.8%) |
| Age (n=811) |  |
| 18-24 | 64 (8.1%) |
| 25-29 | 186 (23.4%) |
| 30-34 | 179 (22.5%) |
| 35-39 | 138 (17.4%) |
| 40-44 | 85 (10.7%) |
| 45-49 | 54 (6.8%) |
| 50+ | 88 (11.1%) |
| Marital Status (n=804) |  |
| Single | 100 (12.4%) |
| Cohabiting | 111 (13.8%) |
| Married | 383 (47.6%) |
| Separated | 30 (3.7%) |
| Divorced | 85 (10.6%) |
| Widow | 95 (11.8%) |
| Occupational Status (n=807) |  |
| Full Time | 614 (76.1%) |
| Part Time | 10 (1.2%) |
| On Leave | 13 (1.6%) |
| Unemployed | 170 (21.1%) |
| Education Level (n=795) |  |
| Nil | 162 (20.4%) |
| Primary | 155 (19.5%) |
| JSS/MSLC | 391 (49.2%) |
| Sec/Tech | 65 (8.2%) |
| Tertiary | 22 (2.8%) |
| Vertical Transmission (n=641) |  |
| Positive | 25 (3.9%) |
| Negative | 50 (7.8%) |
| Positive/Negative | 5 (0.8%) |
| Don't Know | 561 (87.5%) |
| HIV Type (n=453) |  |
| Type I | 411 (90.7%) |
| Type II | 7 (1.5%) |
| Types I/II | 35 (7.7%) |
| HIV Symptoms (n=738) |  |
| 0 | 137 (18.6%) |
| 1 | 160 (21.7%) |
| 2 | 107 (14.5%) |
| 3 | 106 (14.4%) |
| 4 | 88 (11.9%) |
| 5 | 79 (10.7%) |
| 6+ | 61 (8.3%) |
| Other Diseases (n=770) |  |
| 0 | 278 (36.1%) |
| 1 | 276 (35.8%) |
| 2 | 130 (16.9%) |
| 3+ | 86 (11.2%) |
| Sexually Active (n=417) | 346 (66.9%) |
| Contraception Use (n=323) | 130 (40.2%) |
| Contraception Type (n=189) |  |
| Condoms | 151 (79.9%) |
| Injection | 28 (14.8%) |
| Oral contraceptives | 10 (5.3%) |
| Diagnosis Year (n=811) |  |
| 2005 | 6 (0.7%) |
| 2006 | 23 (2.8%) |
| 2007 | 130 (16.0%) |
| 2008 | 162 (20.0%) |
| 2009 | 248 (30.6%) |
| 2010 | 149 (18.4%) |
| 2011 | 72 (8.9%) |
| 2012 | 21 (2.6%) |
| Disclosure (n=746) | 609 (81.6%) |
| WHO Status (n=718) |  |
| I | 181 (25.2%) |
| II | 158 (22.0%) |
| III | 296 (41.2%) |
| IV | 83 (11.6%) |
| On ART (n=761) | 597 (78.4%) |
| Malaria (n=770) | 278 (36.1%) |
| Tuberculosis (n=770) | 40 (5.2%) |
| Sexually Transmitted Infection (n=770) | 57 (10.9%) |
| Respiratory Tract Infection (n=770) | 84 (7.4%) |
| Depression (n=770) | 13 (1.7%) |
| Anemia (n=770) | 66 (8.6%) |
| Hypertension (n=770) | 31 (4.0%) |
| Heart Disease (n=770) | 16 (2.1%) |
| Gastrointestinal Disease (n=770) | 34 (4.4%) |

**Figure A2. Schematic model diagram comparing Option B with Option B Plus**

**Option B**

Pregnant

BF HIV+ Child

BF HIV- Child

Not pregnant, no ART

Not pregnant,

ART

Dead

No antenatal care

Adherent

Not adherent

MTCT

No MTCT

*BF HIV+ Child*

*BF HIV- Child*

MTCT

No MTCT

Adherent

Not adherent

MTCT

No MTCT

CD4 >350

CD4 <350

CD4 >350

CD4 <350

MTCT

No MTCT

CD4 >350

CD4 <350

CD4 >350

CD4 <350

*BF HIV+ Child*

*BF HIV- Child*

Antenatal care

Death

MTCT

No MTCT

*BF HIV+ Child*

BF HIV- Child

No postpartum care

Postpartum care

Death

No postpartum care

Postpartum care

Death

*Not pregnant, no ART*

*Not pregnant, ART*

*Not pregnant, no ART*

*Not pregnant, ART*

*Not pregnant, no ART*

*Not pregnant, ART*

*Not pregnant, no ART*

*Not pregnant, ART*

MTCT

No MTCT

CD4 >350

CD4 <350

CD4 >350

CD4 <350

*Not pregnant, no ART*

*Not pregnant, ART*

*Not pregnant, no ART*

*Not pregnant, ART*

CD4 >350

CD4 <350

CD4 >350

CD4 <350

*Not pregnant, no ART*

*Not pregnant, ART*

*Not pregnant, no ART*

*Not pregnant, ART*

Pregnancy

No pregnancy

Death

CD4 >350

CD4 <350

*Not pregnant, ART*

*Not pregnant, no ART*

*Pregnant*

In care

Not in care

*Not pregnant, no ART*

Pregnancy

No pregnancy

Death

*Pregnant*

*Not pregnant, ART*

*Dead*

*Dead*

*Dead*

*Dead*

*Dead*

*Dead*

**Option B**

**Plus**

Pregnant

BF HIV+ Child

BF HIV- Child

Not pregnant,

ART

Dead

No antenatal care

Adherent

Not adherent

MTCT

No MTCT

*BF HIV+ Child*

*BF HIV- Child*

MTCT

No MTCT

Adherent

Not adherent

MTCT

No MTCT

MTCT

No MTCT

*BF HIV+ Child*

*BF HIV- Child*

Antenatal care

Death

MTCT

No MTCT

*BF HIV+ Child*

BF HIV- Child

No postpartum care

Postpartum care

Death

No postpartum care

Postpartum care

Death

MTCT

No MTCT

*Not pregnant, ART*

*Not pregnant, ART*

Pregnancy

No pregnancy

Death

*Pregnant*

*Not pregnant, ART*

*Dead*

*Dead*

*Dead*

*Dead*

*Dead*

*Not pregnant, ART*

*Not pregnant, ART*

*Not pregnant, ART*

*Not pregnant, ART*

*Not pregnant, ART*

*Not pregnant, ART*

Squares represent decision nodes; circles represent chance events; M represents Markov states, triangles represent terminal nodes. Tracker variables keep track of each woman’s age, number of pregnancies, time between subsequent pregnancies, CD4 count, and prior ART use.

Pregnancy is assumed to last 9 months; breastfeeding is assumed to last 6 months.

Probabilities, costs, and outcomes (QALYs and number of HIV+ children) are assigned to each branch.

MTCT = mother-to-child transmission; BF = breastfeeding; ART = antiretroviral therapy; CD4 = CD4 T-cell count; QALY = quality-adjusted life year.

**Technical Appendix References**

1. Ghana Health Service. PMTCT Annual Report. Kumasi, Ghana: National AIDS/STI Control Programme, Office KR; 2011.

2. Business Leadership Council. A Business Case for Options B and B+ to Eliminate Mother to Child Transmission of HIV by 2015. 2012.

3. United Nations. The Impact of AIDS. United Nations, Department of Economic and Social Affairs PD; 2004.
